# Contracted Cicatrix: Autoplastic Operation

**Published:** 1846-07

**Authors:** John Fife


*Contracted Cicatrix: Autoplastic Operation.* By Sir John Fife.— An operation for contracted cicatrix, on the plan proposed by Professor Mütter, of the United States, has been recently performed in the Newcastle-on-Tyne Infirmary. The patient was a young girl, Eliza Goodchild, seven years of age. The contractions followed a burn of the throat, and prevented the head being raised, and the under lip being closed.

Sir John Fife had operated on the case once before, by simply dividing the adhesions, and fixing back the head; but after cicatrisation the integuments again became contracted, though not, as before, binding the chin down to the breast.

The operation was performed on the morning of the 7th of April. Sir John Fife commenced by making an incision entirely across the throat, followed by a perpendicular incision at each end of it; he then dissected between the integuments and the cartilages of the larynx, so as to admit of an extensive separation of the contracted integuments. A considerable portion of integument was now dissected from above the right clavicle, turned at the right perpendicular incision, and fixed across the throat by seven or eight stitches. The right external jugular vein was exposed, but not wounded; a branch from the right superior thyroid artery bled freely. Some lint was placed round the neck, covered with simple ointment. Four hours after the operation there was some secondary hæmorrhage; the dressings were removed, and another arterial branch near the former was tied. The integument placed across the throat looked slightly livid, but evidently retained its vitality.

During the subsequent progress of the case, one-half of the transplanted portion of integument lost its vitality, but the condition of the patient was nevertheless greatly improved.

The result of the foregoing operation appears to confirm the propriety of a suggestion made by Professor Mütter, that when the contraction is extensive a portion of the skin intended to supply the vacancy should be taken from either side, thus forming a double flap to meet in the centre. This method was actually adopted by Mr. Carden, of Worcester, in a case published in the twelfth volume of "The Transactions of the Provincial Medical and Surgical Association," the operation having been performed before Professor Mütter's paper was published, and, indeed, before that able surgeon had himself employed the autoplastic operation in these cases. As the volume of the "Transactions" containing the report of Mr. Carden's case is not in the possession of those of our readers who have joined the Association since its publication, and the case is itself of great interest in its bearings on the subject, the following abstract of it may probably be acceptable.

Mary Ann Barnett, aged 14, was admitted into the Worcester Infirmary, September 9th, 1839, with contracted cicatrix after a burn, which had occurred seven years previously. The movements of the head were greatly restricted; the mouth remained permanently open, the tongue protruded, the lower incisors projected horizontally, and there was constant salivation. On attempting to raise the head, the eyelids were drawn considerably downwards. The patient, as well as her friends, being anxious to have something done for her relief, a consultation was held upon the case, and with the consent of his colleagues at the Infirmary, Mr. Carden performed the following operation:—

The patient being placed on a well-cushioned table, with her head and shoulders somewhat elevated, the operation was commenced by carefully gathering up the cicatrix from below the left ear to the top of the sternum, between the fingers and the thumb of the left hand, which allowed of the whole of that side to be transfixed and divided at a stroke; the same was repeated on the right side, and a short cut over the top of the sternum connected the two incisions. In this manner the whole transverse extent of the cicatrix was rapidly divided, the wound terminating in sound skin on each side. The chin was then drawn upwards by an assistant, and every tense band of cicatrix successively divided by repeated strokes of the scalpel, until the head was released into nearly its natural position. By this mode of dissection, although nothing had been removed, the hiatus produced was very great, and extended from the chin and edge of the lower jaw to below the upper border of the sternum, exposing the greater part of both sterno-mastoid muscles, and external jugular and thyroid veins, the latter being particularly large and prominent. The quantity of blood lost was very trifling, scarcely requiring the torsion forceps. As soon as all bleeding had ceased, Mr. Carden proceeded to select a portion of sound skin on each side, about three inches long, and two and a half wide; these were raised and detached, except at their junction with the outer edges of the wound, and brought together across the centre of the neck, and there united by hare-lip needles. The side wounds left by the flaps were then brought together, and the exposed parts covered with lint. The flaps were carefully supported by adhesive plaster, leaving apertures for the points of the needles, and the whole of the wound and surrounding integuments were well supported by long plasters and bandages.

The operation, which was severe and necessarily protracted, was borne with great fortitude, and without fainting. The needles were withdrawn two days after the operation; the dressings were not removed until the sixth day, when the flaps were found to have retained their position; but the upper border of each, being composed of old cicatrix, had perished, diminishing the breadth of each to less than two inches. The complete healing of the wound occupied nearly twelve months, during which time various contrivances were had recourse to for keeping the head in the erect position; but the bodily and mental suffering was so great, each time the wound was dressed, from these repeated stretchings, that they were altogether discontinued.

She was made out-patient in May, 1840, and in November following presented the following appearances:—Wound healed; position and movements of the head greatly improved; can close the mouth, retain the saliva, and articulate distinctly; teeth regaining their natural position. A narrow cord has sprung up between the flaps, which threatens to draw down the centre of the lower lip, and also to prevent the further expansion of the flaps, which has hitherto been steadily going on and forming the most satisfactory feature of the case.

This band was divided by a curved bistoury, and pressure and further extension enjoined; but from that time she avoided attendance at the hospital, and neglected all directions. No more was seen of her until October 23d, 1843. She had then become stout in person; the teeth were quite upright; the flaps measured three inches on the right side, and two inches and three quarters on the left, from above downwards; but there was an increased contraction in the central cord. This she consented to have divided and separated from the flaps, which would then, it was thought, be permanently united, and her appearance considerably improved.—***Provin. Med. and Surg. Journal.***

